# A cutset-type kernel possibilistic fuzzy c-means method for robust image segmentation

**DOI:** 10.3389/frai.2026.1886592

**Published:** 2026-07-15

**Authors:** Jintao Wang, Zhenxing Xu, Kang Feng, Liting Zhang

**Affiliations:** 1 College of Computing and Artificial Intelligence, Wanjiang University of Technology, Maanshan, Anhui, China; 2 College of Computer Science and Software Engineering, Hohai University, Nanjing, Jiangsu, China; 3 College of Systems Engineering, National University of Defense Technology, Changsha, Hunan, China

**Keywords:** cutset-type clustering, image segmentation, kernel possibilistic fuzzy c-means, robust clustering, unsupervised segmentation

## Abstract

**Background:**

Robust image segmentation remains difficult for lightweight unsupervised clustering when nonlinear class overlap and sparse noise contamination occur together.

**Methods:**

This study proposes and evaluates a cutset-type kernel possibilistic fuzzy c-means method (C-KPFCM), combining Gaussian-kernel distance modeling with cutset-based correction of possibilistic typicalities. The kernel term changes the distance geometry for nonlinear structure, while the cutset operation suppresses non-winning typical values during center estimation.

**Results:**

On the three reconstructed complex-background cases, C-KPFCM obtains the best average result among the tested classical fuzzy clustering methods, but the gain over the closest cutset-only baseline is small and not statistically reliable under an exact sign test. A 90-instance stress test further shows that C-KPFCM is clearly better than K-means, PFCM, and KPFCM, but not generally better than C-PFCM under broader disturbance mixtures. Additional noise, parameter-sensitivity, repeated-run, runtime, and real-image pilot analyses support a restrained conclusion.

**Conclusion:**

Cutset correction is the empirically dominant robustness mechanism, while kernelization is a conditional complement whose cost is justified only when nonlinear geometry is expected to matter. Code and reproducibility scripts are available at: jtwang-AI/Image-Segmentation-C-KPFCM.

## 1 Introduction

Image segmentation is a foundational step in engineering workflows such as industrial inspection, remote-sensing interpretation, biomedical image analysis, and machine-vision preprocessing. In these applications, practitioners often encounter weak boundaries, complex background interference, sensor noise, and limited annotated data. Although large supervised and foundation-style segmentation models have substantially improved semantic segmentation quality, compact unsupervised methods remain useful when training data, computational resources, or deployment transparency are constrained.

Fuzzy c-means (FCM) is a representative lightweight segmentation method because it models boundary ambiguity through soft assignments while keeping the optimization simple (Bezdek et al., 1984). Its Euclidean prototype assumption, however, is weak when image classes are nonlinear, non-convex, or strongly contaminated by background pixels. These limitations become especially visible in images with intensity inhomogeneity, local corruption, or cluttered boundaries. Prior work has therefore extended fuzzy clustering through sparse regularization, alternative distance measures, local-neighborhood constraints, kernel formulations, and possibilistic typicality terms (Qiu et al., 2015; Zhao et al., 2015, 2014; Krinidis and Chatzis, 2010; Wu and Cao, 2020; Pal et al., 2005; Anderson et al., 2010).

In parallel, recent segmentation research has shifted toward transformer-based, open-vocabulary, and promptable systems, including Mask2Former, TopFormer, Panoptic SegFormer, Segment Anything, and later open-vocabulary segmentation pipelines (Cheng et al., 2022; Zhang et al., 2022; Li et al., 2022; Kirillov et al., 2023; Xie et al., 2024). These approaches are powerful, but they usually rely on large training corpora, heavier models, and more complex deployment pipelines. This does not remove the need for classical objective-level segmentation methods; instead, it clarifies their role. They are not competitors to foundation models in broad semantic understanding, but practical alternatives for small, reproducible, and low-compute segmentation tasks.

This paper focuses on robust image segmentation under three coupled difficulties: nonlinear class overlap, sparse local contamination, and complex background interference. These difficulties stress different parts of a clustering objective. Nonlinear overlap weakens Euclidean center-based separation because pixels from different regions can have similar centroids or curved manifolds in feature space. Sparse contamination weakens membership-only updates because corrupted pixels can still contribute to center estimation. Possibilistic typicality reduces this pressure, but without inter-class suppression a contaminated pixel may remain moderately typical for several clusters. We therefore revisit a cutset-type kernel possibilistic fuzzy c-means model (C-KPFCM) and frame the paper around a testable mechanism question: after cutset correction has suppressed unreliable typicalities, does kernelization add measurable benefit?

The main contributions are as follows:

1. It formulates C-KPFCM as an explicit combination of kernelized distance modeling and cutset-based typicality correction, separating representation geometry from contamination control.
2. It provides an ablation-centered evaluation showing that cutset correction is the dominant robustness mechanism, while kernelization contributes only a small conditional complement.
3. It adds statistical reliability checks, an expanded 90-instance disturbance benchmark, runtime details, and real-image pilot evidence to define the method's practical boundary instead of overstating broad segmentation superiority.

## 2 Related work

### 2.1 Fuzzy clustering for image segmentation

Classical fuzzy clustering methods assign pixels to clusters through continuous memberships and are therefore naturally suited to ambiguous image boundaries. FCM remains a common baseline because of its simplicity and low computational cost (Bezdek et al., 1984). Subsequent work has improved fuzzy segmentation by modifying the distance criterion, incorporating local image context, or adding robustness terms. Mahalanobis-distance and neighborhood-weighted variants attempt to address non-spherical structure or spatial coherence (Zhao et al., 2015, 2014), while local-information FCM improves robustness under noise by using neighborhood statistics (Krinidis and Chatzis, 2010). Other robust fuzzy clustering methods introduce sparse, ambiguous-set, quantum-inspired, or reliability-aware formulations for difficult image conditions (Qiu et al., 2015; Malhotra and Jha, 2021; Singh and Bose, 2021b,a; Pan et al., 2021).

Recent ambiguous and kernel-distance clustering studies for COVID-19 CT analysis are especially relevant because they also use clustering objectives to handle medical-image ambiguity and weak boundary evidence (Singh and Bose, 2021b,a; Singh and Huang, 2024a,b). The present work differs in two ways. First, it studies a cutset-type possibilistic correction rather than an ambiguous-set decision rule or edge detector. Second, it evaluates the kernel term as an ablation against a cutset-only baseline, so the contribution is framed as a mechanism test rather than a claim that kernelization alone dominates existing clustering segmentation methods.

These variants reveal a common trade-off. Spatially constrained and local-information methods can suppress isolated noise, but their performance depends on neighborhood assumptions that may blur thin or weak boundaries. Distance-metric and kernelized methods can improve nonlinear separability, but they do not by themselves decide which atypical pixels should stop influencing multiple centers. This distinction motivates treating distance modeling and evidence suppression as separate mechanisms in the experiments rather than presenting their combination as automatically superior.

### 2.2 Possibilistic and cutset-type clustering

Possibilistic clustering addresses a different failure mode. In standard fuzzy clustering, memberships are constrained to sum to one for each sample, which can force outliers or corrupted pixels to contribute too strongly to some cluster. Possibilistic typicality relaxes this behavior by measuring how representative a sample is of a cluster independently from the partition constraint. PFCM combines fuzzy membership and possibilistic typicality to reduce the coincident-cluster problem of PCM while retaining noise resistance (Pal et al., 2005; Anderson et al., 2010). Cutset-type possibilistic methods further sharpen the typicality structure by retaining the winning typicality when it exceeds a threshold and suppressing competing non-winning typicalities (Yu and Fan, 2018; Fan and Gao, 2021). This is the main robustness idea used in the present method.

### 2.3 Kernelized clustering and modern segmentation

Kernelized clustering methods address nonlinearity by replacing Euclidean distance with an implicit feature-space distance. Kernel PFCM and related kernel fuzzy methods are useful when clusters are poorly represented by linear prototypes in the original feature space (Lu and Xiong, 2006; Wu and Zhou, 2005; Wu and Cao, 2020). This motivation differs from modern deep segmentation, where nonlinearity is learned through high-capacity networks. Transformer and foundation-style models now define the state of the art for many supervised, weakly supervised, and open-vocabulary segmentation tasks (Cheng et al., 2022; Ding et al., 2022; Du et al., 2022; Shi et al., 2023; Zhang et al., 2023; Ma et al., 2023; Li et al., 2023; Hu et al., 2024; Sung et al., 2024; Zhu et al., 2024; Lee et al., 2025; Lu et al., 2025; Ulger et al., 2025; Qiu et al., 2025). The present work is complementary to that line: it studies a compact unsupervised objective that can be inspected, rerun, and deployed without a large training pipeline.

The comparison scope is therefore deliberately narrower than a modern semantic-segmentation benchmark. We do not claim state-of-the-art performance against recent deep models. Instead, the experiments ask whether the cutset and kernel components improve a classical unsupervised clustering family under controlled disturbance modes. The absence of recent lightweight deep baselines remains a limitation, and the real-image pilots are used only to test transfer of the mechanism rather than to establish broad superiority.

## 3 Materials and methods

### 3.1 Problem setting

Let $X={\left\{x_{i}\right\}}_{i=1}^{n}$ denote image pixels or pixel-level feature vectors, where $x_{i}\in {\mathbb{R}}^{d}$. The goal is to partition the image into *c* regions by estimating cluster centers $V={\left\{v_{k}\right\}}_{k=1}^{c}$, fuzzy memberships *U* = [*u*_*ki*_], and possibilistic typicalities *T* = [*t*_*ki*_]. The memberships satisfy 0 ≤ *u*_*ki*_ ≤ 1 and $\overset{c}{\underset{k=1}{\sum }}u_{ki}=1$, while the typicalities satisfy 0 ≤ *t*_*ki*_ ≤ 1 without a cross-cluster sum constraint.

The proposed method combines two robustness components. The kernel component changes the distance geometry so that nonlinear class structure can be represented more effectively. The cutset component changes the typicality pattern so that unreliable non-winning cluster responses are prevented from influencing multiple centers.

### 3.2 Design motivation and data flow

**Figure 1:**
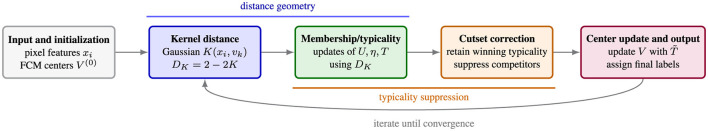
Data flow of C-KPFCM. Kernel mapping enters through the distance computation, while cutset correction enters after the typicality update and before center estimation.

The design separates representation mismatch from contamination control, and Figure 1 summarizes the data flow of the proposed method. Kernel mapping is used because Euclidean prototypes can be inadequate when foreground and background pixels form non-convex or centroid-sharing structures. Cutset correction is used because possibilistic typicalities can remain nonzero for multiple clusters, allowing ambiguous or corrupted samples to influence several center updates. Combining the two mechanisms is therefore not intended to create a new optimizer; it creates a testable decomposition in which the kernel term changes the distance *D*_*K*_ and the cutset term changes which typicalities contribute to Equation 7.

### 3.3 Kernelized PFCM objective

The C-KPFCM objective is given in Equation 1. The Gaussian kernel in Equation 2 induces the feature-space distance in Equation 3.


$$\begin{array}{l} J\left(U,T,V\right)=\overset{n}{\underset{i=1}{\sum }}\overset{c}{\underset{k=1}{\sum }}\left(au_{ki}^{m}+bt_{ki}^{p}\right)∥\Phi \left(x_{i}\right)-\Phi \left(v_{k}\right){∥}^{2}\\ \;+\overset{c}{\underset{k=1}{\sum }}\eta_{k}\overset{n}{\underset{i=1}{\sum }}{\left(1-t_{ki}\right)}^{p}, \end{array}$$ (1)

where *m* > 1 and *p* > 1 are fuzzification parameters, *a* and *b* balance membership and typicality terms, η_*k*_ is a positive scale parameter, and Φ(·) is the implicit kernel mapping.

Using a Gaussian kernel,


$$\begin{array}{l} K\left(x_{i},v_{k}\right)=\exp\left(-\frac{∥x_{i}-v_{k}{∥}^{2}}{\sigma^{2}}\right), \end{array}$$ (2)

with *K*(*x, x*) = 1, the feature-space distance becomes


$$\begin{array}{l} D_{K}\left(x_{i},v_{k}\right)=∥\Phi \left(x_{i}\right)-\Phi \left(v_{k}\right){∥}^{2}\\ \;=K\left(x_{i},x_{i}\right)+K\left(v_{k},v_{k}\right)-2K\left(x_{i},v_{k}\right)\\ \;=2-2K\left(x_{i},v_{k}\right). \end{array}$$ (3)

Minimizing Equation 1 under the membership constraint gives the following updates:


$$\begin{array}{l} u_{ki}=\frac{{\left[D_{K}\left(x_{i},v_{k}\right)\right]}^{-\frac{1}{m-1}}}{\sum_{j=1}^{c}{\left[D_{K}\left(x_{i},v_{j}\right)\right]}^{-\frac{1}{m-1}}}, \end{array}$$ (4)

$$\begin{array}{l} \eta_{k}=\frac{\sum_{i=1}^{n}u_{ki}^{m}D_{K}\left(x_{i},v_{k}\right)}{\sum_{i=1}^{n}u_{ki}^{m}}, \end{array}$$ (5)

$$\begin{array}{l} t_{ki}={\left[1+{\left(\frac{bD_{K}\left(x_{i},v_{k}\right)}{\eta_{k}}\right)}^{\frac{1}{p-1}}\right]}^{-1}, \end{array}$$ (6)

$$\begin{array}{l} v_{k}=\frac{\sum_{i=1}^{n}\left(au_{ki}^{m}+bt_{ki}^{p}\right)K\left(x_{i},v_{k}\right)x_{i}}{\sum_{i=1}^{n}\left(au_{ki}^{m}+bt_{ki}^{p}\right)K\left(x_{i},v_{k}\right)}. \end{array}$$ (7)

Small numerical constants are used in implementation to avoid division by zero when kernel distances become extremely small.

### 3.4 Cutset correction

For each sample *x*_*i*_, Equation 8 identifies the cluster with the largest typicality:


$$\begin{array}{l} q\left(i\right)=\arg\underset{1\leq k\leq c}{\max}t_{ki}. \end{array}$$ (8)

Given a threshold beta in [0, 1], the corrected typicality is defined in Equation 9 as follows:


$$\begin{array}{l} {\tilde{t}}_{ki}=\{\begin{array}{ll} t_{ki}, & \max_{j}t_{ji}\leq \beta ,\\ t_{ki}, & \max_{j}t_{ji}>\beta \text{ and }k=q(i),\\ 0, & \max_{j}t_{ji}>\beta \text{ and }k\neq q(i). \end{array} \end{array}$$ (9)

The rule preserves all typicalities for uncertain samples whose maximum typicality is below the cutset threshold. Once a sample has a clear winning typicality, only the winner is retained and all competing non-winning typicalities are suppressed. The center update in Equation 7 then uses ${\tilde{t}}_{ki}$ in place of *t*_*ki*_.

This correction is the main distinction between KPFCM and C-KPFCM. It introduces inter-class competition into the possibilistic component without removing the fuzzy membership constraint. As a result, corrupted or ambiguous pixels are less likely to influence several clusters at the same time.

### 3.5 Algorithm

Algorithm 1 summarizes the complete C-KPFCM iteration and final assignment procedure.

Algorithm 1 **C-KPFCM for image segmentation.**

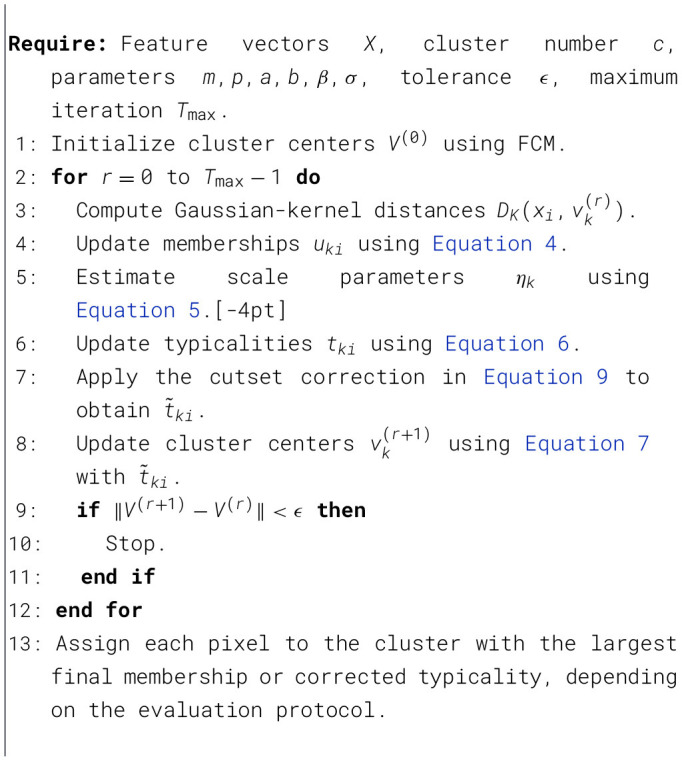

### 3.6 Computational complexity and scalability

Let *n* be the number of pixels or feature vectors, *c* the number of clusters, *d* the feature dimension, and *T* the number of iterations. Table 1 summarizes the per-iteration operations, time complexity, and memory complexity of the compared clustering objectives. FCM requires pairwise sample–center distance computation and center updates, giving time complexity *O*(*Tncd*) and memory complexity *O*(*nc*) for the membership matrix. PFCM keeps the same asymptotic time and memory order, but adds typicality and scale-parameter updates. KPFCM and C-KPFCM also remain *O*(*Tncd*) for the Gaussian radial basis kernel used here because only sample–center kernel values are computed; no full *n*×*n* Gram matrix is formed. Their practical runtime is nevertheless higher because every iteration evaluates exponentials and uses kernel-weighted center updates.

**Table 1:** Computational profile of the compared clustering objectives.

Method	Main per-iteration operations	Time complexity	Memory complexity
K-means	Hard assignment and center update	*O*(*Tncd*)	*O*(*n*+*cd*)
FCM	Distances, memberships, centers	*O*(*Tncd*)	*O*(*nc*+*cd*)
PFCM/C-PFCM	FCM plus typicalities and η_*k*_	*O*(*Tncd*)	*O*(*nc*+*cd*)
KPFCM/C-KPFCM	Kernel distances plus typicalities	*O*(*Tncd*)	*O*(*nc*+*cd*)

Constants differ in practice because kernel methods evaluate exponentials and possibilistic methods maintain typicalities.

The cutset step adds only *O*(*Tnc*) time and no additional asymptotic memory beyond the typicality matrix. Therefore, the additional cost of C-KPFCM relative to C-PFCM is dominated by the Gaussian-kernel distance computation, not by the cutset rule. In deployment, this means the kernel component is justified when nonlinear color–spatial geometry is expected to affect the segmentation, but C-PFCM is the more economical choice when sparse contamination is the dominant difficulty and Euclidean geometry is adequate.

### 3.7 Experimental design and implementation

The experiments test whether C-KPFCM improves image segmentation accuracy relative to conventional fuzzy clustering baselines under engineering-relevant disturbances. Three reproducible synthetic image cases are used, denoted CAO, GU, and WEI. They simulate complex background interference, nonlinear class overlap, weak boundaries, and local contamination pixels. The compared methods are FCM, PFCM, KPFCM, C-PFCM, and C-KPFCM. FCM and PFCM are classical baselines. KPFCM and C-PFCM serve as ablations because they separately represent the kernel-only and cutset-only parts of the full method.

In response to the concern that three synthetic cases are insufficient for statistical interpretation, an additional reviewer-requested stress benchmark is included. It combines the three base cases with six disturbance settings: clean, Gaussian noise, impulse noise, multiplicative speckle noise, mixed Gaussian–impulse noise, and texture-plus-occlusion corruption. Five random seeds are used for each case–disturbance pair, resulting in 90 evaluated instances. This expanded benchmark also includes a simple K-means baseline as a lightweight hard-clustering reference. It is still not a replacement for large real-image semantic segmentation benchmarks, but it provides a broader controlled test of the mechanism under multiple degradation modes.

All methods are applied to the same input images and evaluated against the same reference segmentations. Unless otherwise stated, the reported configuration uses *m* = 2, *p* = 2, *a* = 1, *b* = 1, β = 0.5, σ = 0.26, a maximum of 80 iterations, and a convergence tolerance of 10^−5^. FCM initialization is used for the possibilistic and kernelized variants to reduce sensitivity to poor starting centers.

The synthetic cases contain compact and curved structures, shared or nearby centroids, cluttered backgrounds, and sparse adversarial pixels. Their role is to isolate the difficulty modes that the proposed objective is designed to address. They are controlled stress tests rather than a substitute for broad natural-image evaluation.

All reported runtime values were obtained on macOS 26.5 with an Apple M5 CPU and 16 GB unified memory. The implementation used Python 3.13.3 with NumPy, Pillow, and Matplotlib. The real-image pilots resized images to 96 × 96 before clustering; the Oxford-IIIT Pet feature vector used RGB values plus spatial coordinates weighted by 0.20, and the Weizmann pilot used the same feature form with spatial weight 0.12. Absolute runtimes should therefore be interpreted as implementation-specific reproducibility information rather than hardware-independent performance claims.

### 3.8 Evaluation metrics

Segmentation accuracy (SA), defined in Equation 10, is used as the primary synthetic-benchmark metric:


$$\begin{array}{l} SA=\frac{\sum_{i=1}^{c}|A_{i}\cap C_{i}|}{\sum_{i=1}^{c}|C_{i}|}, \end{array}$$ (10)

where *A*_*i*_ denotes the set of pixels assigned to class *i* by a clustering algorithm and *C*_*i*_ denotes the corresponding reference region. A larger SA indicates closer agreement with the reference segmentation.

For the real-image pilots, Dice and intersection-over-union (IoU) are also reported because those datasets are evaluated as foreground-background segmentation tasks.

## 4 Results

### 4.1 Main quantitative comparison

**Figure 2:**
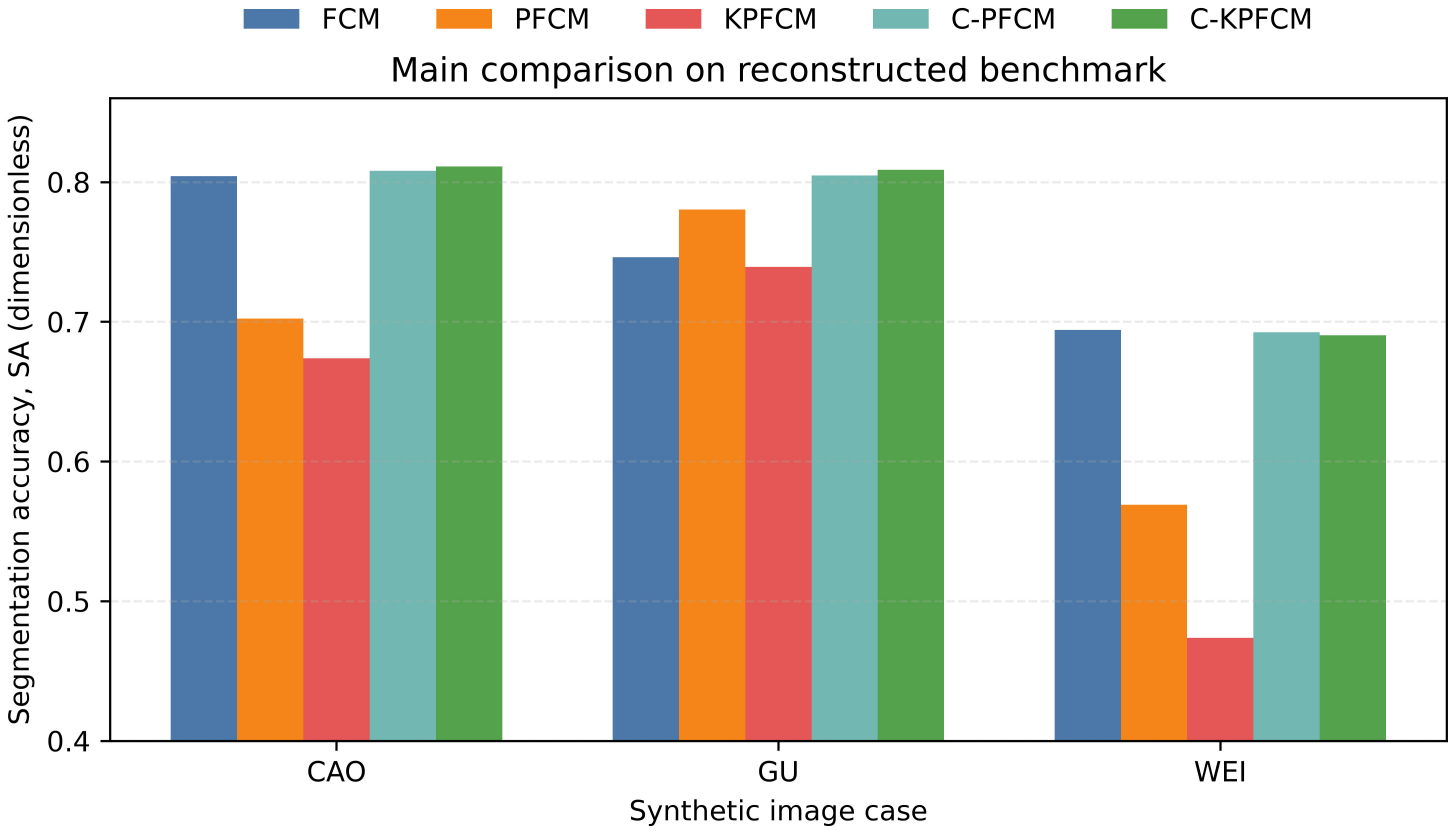
Main quantitative comparison across the CAO, GU, and WEI cases. C-KPFCM obtains the highest average SA and the best result on two of the three cases.

Table 2 reports the main synthetic comparison. Figure 2 visualizes the same main comparison across CAO, GU, and WEI. C-KPFCM achieves the highest SA on CAO and GU, while C-PFCM is marginally better on WEI. Averaged over all cases, C-KPFCM obtains the best overall SA of 0.7699.

**Table 2:** Segmentation accuracy of different clustering algorithms on synthetic complex-background image cases.

Case	FCM	PFCM	KPFCM	C-PFCM	C-KPFCM
CAO	0.8041	0.7023	0.6737	0.8079	0.8110
GU	0.7461	0.7802	0.7393	0.8047	0.8086
WEI	0.6942	0.5690	0.4736	0.6924	0.6902
Average	0.7482	0.6838	0.6289	0.7683	0.7699

The result supports a mechanism-level interpretation. KPFCM alone is weaker than both cutset-based variants, showing that kernelization by itself is not the dominant source of robustness. C-PFCM is already strong, which indicates that suppressing non-winning typicalities is the key improvement. C-KPFCM adds a small positive gain over C-PFCM on average, suggesting that kernelization becomes useful after the typicality structure has been stabilized.

Table 3 makes the same pattern explicit. The gain over PFCM is substantial, the gain over FCM is moderate, and the gain over C-PFCM is small. The full method is therefore best described as a cutset-dominated robust clustering method with a kernelized complement.

**Table 3:** Average performance gain of C-KPFCM over reference methods on the synthetic benchmark.

Comparison	Absolute gain	Relative gain
C-KPFCM vs. FCM	0.0218	2.91%
C-KPFCM vs. PFCM	0.0861	12.59%
C-KPFCM vs. C-PFCM	0.0016	0.21%

Table 4 addresses the reliability of the ablation result. Because the synthetic benchmark contains only three cases, none of the paired comparisons can establish statistical significance. This is especially important for the closest baseline: the C-KPFCM–C-PFCM difference is only 0.0016 on average, with one of the three cases favoring C-PFCM. The correct interpretation is therefore not that kernelization is proven superior, but that the reconstructed evidence strongly favors cutset correction and only weakly suggests a conditional kernel benefit.

**Table 4:** Paired reliability check for C-KPFCM against reference methods across the three synthetic cases.

Comparison	Mean paired difference	SD of difference	W/T/L	Sign-test *p*
C-KPFCM–FCM	0.0218	0.0357	2/0/1	1.0000
C-KPFCM–PFCM	0.0861	0.0504	3/0/0	0.2500
C-KPFCM–KPFCM	0.1411	0.0737	3/0/0	0.2500
C-KPFCM–C-PFCM	0.0016	0.0033	2/0/1	1.0000

The sign test is exact and two-sided; W/T/L denotes the number of cases where C-KPFCM wins, ties, or loses.

### 4.2 Expanded reviewer-requested stress benchmark

**Figure 3:**
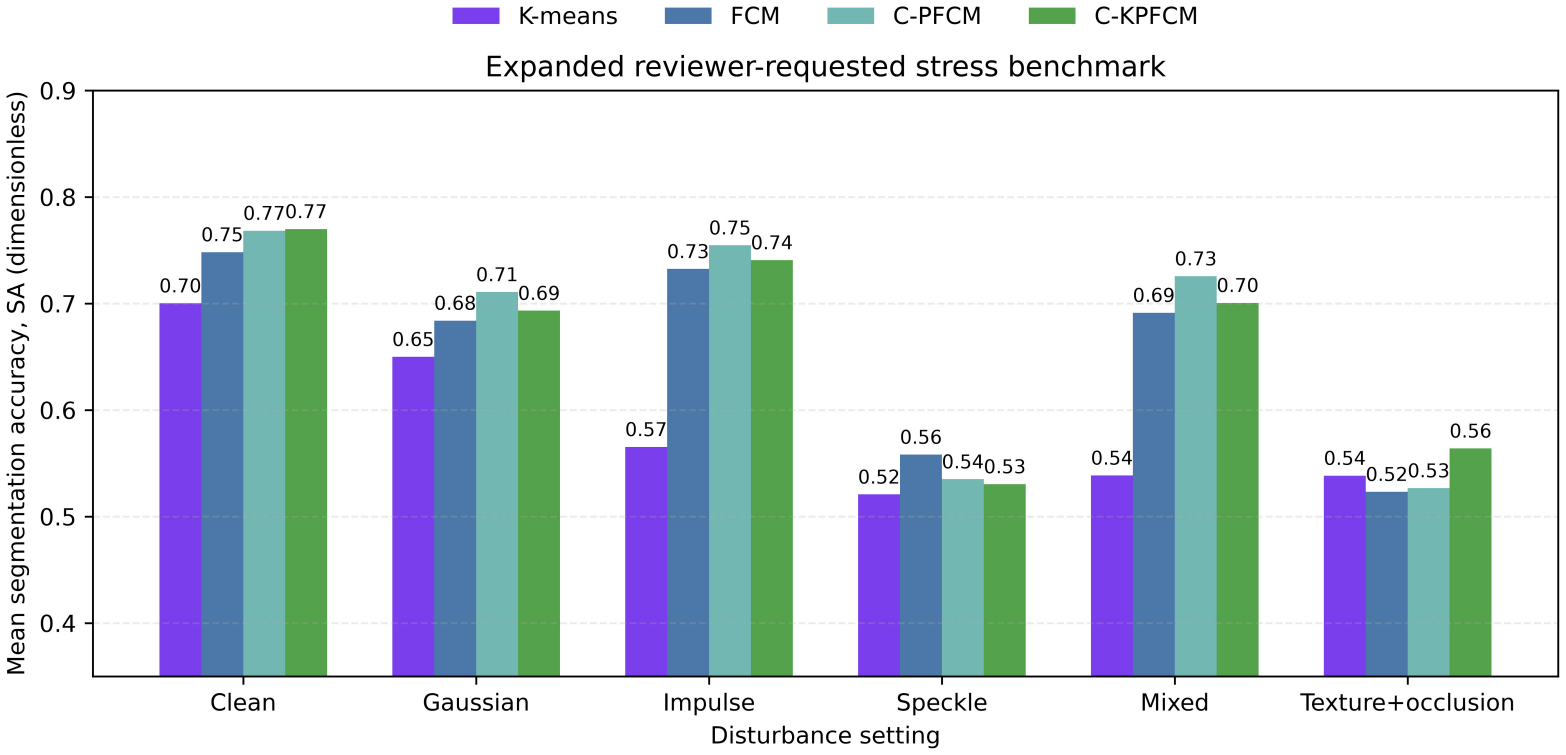
Expanded reviewer-requested stress benchmark across six disturbance settings. The figure emphasizes that the full kernelized method is not uniformly superior; it is most useful in clean nonlinear geometry and texture-occlusion cases, while C-PFCM is often stronger for local noise corruption.

To address the limited sample size of the initial synthetic benchmark, the expanded stress benchmark evaluates 90 controlled instances: three base cases, six disturbance settings, and five random seeds for each case–disturbance pair. Table 5 reports the pooled performance across all expanded instances. Figure 3 visualizes the mean SA values across the six disturbance settings. C-KPFCM remains clearly stronger than K-means, PFCM, and KPFCM, but it no longer exceeds C-PFCM after the disturbance space is broadened. The overall mean SA is 0.6665 for C-KPFCM and 0.6701 for C-PFCM. This result directly limits the practical claim: the cutset mechanism is robustly useful, whereas the Gaussian-kernel term should be treated as a conditional addition rather than a default improvement.

**Table 5:** Expanded 90-instance reviewer-requested stress benchmark.

Method	*n*	SA mean	SA std	Time mean (s)
K-means	90	0.5855	0.0806	0.0135
FCM	90	0.6562	0.1004	0.0536
PFCM	90	0.5742	0.0969	0.0490
KPFCM	90	0.5677	0.0963	0.0604
C-PFCM	90	0.6701	0.1111	0.0548
C-KPFCM	90	0.6665	0.1048	0.0792

Values are pooled over clean, Gaussian, impulse, speckle, mixed-noise, and texture-occlusion settings.

The paired sign tests in Table 6 show the same pattern. C-KPFCM wins decisively against K-means, PFCM, and KPFCM, but not against FCM or C-PFCM. Against C-PFCM, C-KPFCM wins on 25 instances, ties on one, and loses on 64, so the expanded test favors the cutset-only variant under this broader disturbance mixture. This is an important negative result rather than a failure of the evaluation: it clarifies that kernelization adds computational cost and may not improve performance when the dominant problem is local corruption rather than nonlinear separability.

**Table 6:** Paired checks for C-KPFCM on the expanded 90-instance stress benchmark.

Reference	Mean difference	SD of difference	W/T/L	Sign-test *p*
K-means	0.0810	0.0851	75/0/15	< 0.0001
FCM	0.0103	0.0431	42/1/47	0.6718
PFCM	0.0922	0.0639	84/0/6	< 0.0001
KPFCM	0.0987	0.0669	87/0/3	< 0.0001
C-PFCM	–0.0037	0.0318	25/1/64	< 0.0001

Mean difference is C-KPFCM minus the reference method. Ties are excluded from the exact two-sided sign test.

Table 7 breaks the expanded benchmark down by disturbance type. The cutset-based methods are strongest in the clean, Gaussian, impulse, and mixed-noise settings. C-KPFCM is best only in the clean setting and in the texture-plus-occlusion setting, where nonlinear geometry and clutter are more prominent. C-PFCM is stronger under Gaussian, impulse, and mixed noise, which is consistent with the interpretation that typicality suppression is the dominant mechanism under local corruption.

**Table 7:** Mean SA by disturbance type on the expanded benchmark.

Disturbance	K-means	FCM	C-PFCM	C-KPFCM
Clean	0.7002	0.7482	0.7683	0.7699
Gaussian	0.6499	0.6839	0.7107	0.6932
Impulse	0.5653	0.7324	0.7546	0.7407
Speckle	0.5207	0.5581	0.5350	0.5304
Mixed	0.5385	0.6913	0.7256	0.7006
Texture+occlusion	0.5383	0.5232	0.5266	0.5639

The table reports the lightweight hard-clustering reference and the most relevant fuzzy/cutset baselines.

### 4.3 Ablation and qualitative comparison

**Figure 4:**
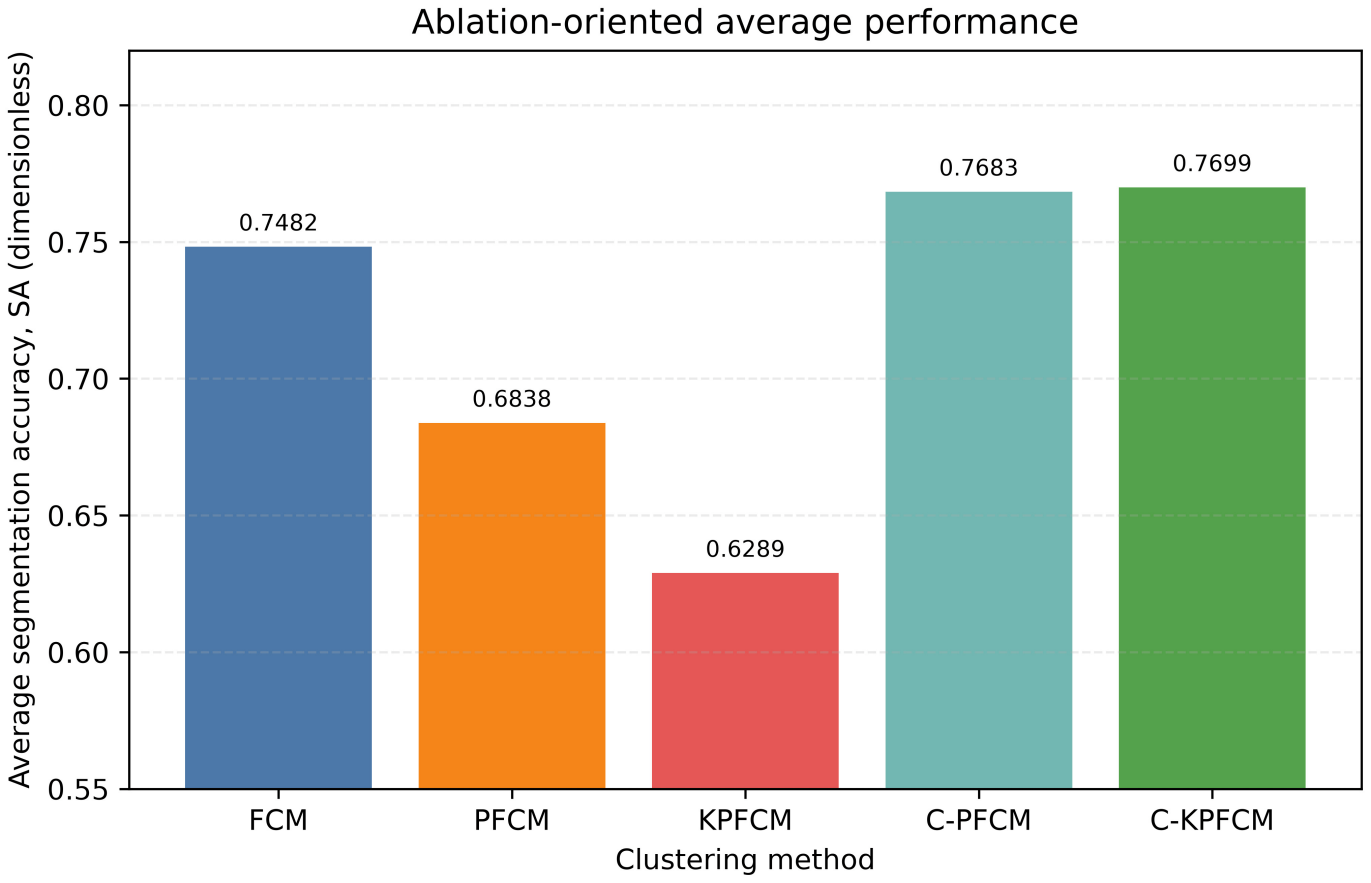
Ablation-oriented average performance. The cutset-based variants clearly outperform the kernel-only variant, while the full C-KPFCM model slightly improves the average over C-PFCM.

Figure 4 reinforces the central ablation result. If the main difficulty is corrupted or ambiguous pixels that retain non-negligible typicalities for several clusters, cutset correction directly reduces this interference. Kernel distance is useful when nonlinear geometry is part of the segmentation difficulty, but the present evidence does not show a statistically reliable improvement over the cutset-only variant.

### 4.4 Noise robustness and compound disturbances

**Figure 5:**
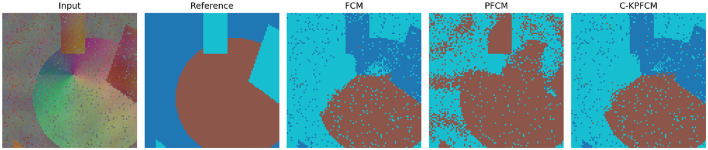
Representative qualitative comparison on the reconstructed CAO case. The visual pattern matches the quantitative benchmark: C-KPFCM suppresses more clutter than FCM and avoids some fragmented responses visible in possibilistic variants without cutset correction.

**Figure 6:**
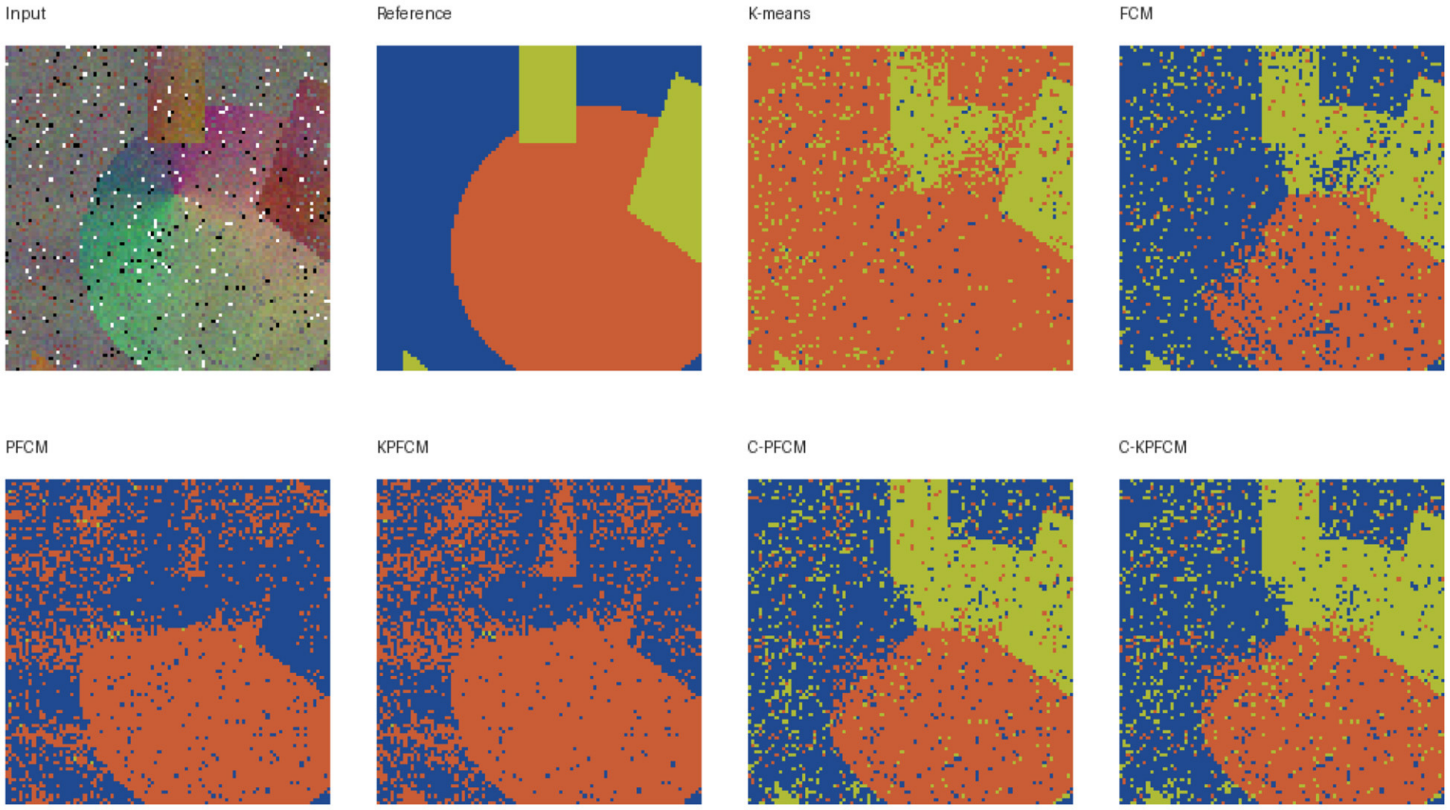
Expanded qualitative comparison on a mixed-noise CAO instance. All six compared methods are shown with the input and reference mask. The visual result illustrates why the quantitative conclusions are restrained: cutset-based variants reduce some fragmented typicality responses, but C-KPFCM does not uniformly dominate C-PFCM under mixed corruption.

**Figure 7:**
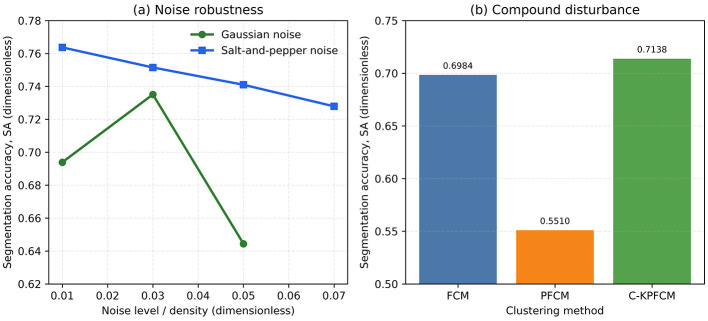
Robustness evidence under recovered noise settings. **(a)** C-KPFCM remains comparatively stable under sparse salt-and-pepper corruption, while Gaussian corruption is more sensitive to noise level. **(b)** In the recovered compound-disturbance setting, C-KPFCM achieves the strongest SA among the three available methods.

Additional robustness experiments are conducted under Gaussian noise and salt-and-pepper noise. Figures 5, 6 provide representative qualitative comparisons, and Figure 7 summarizes the recovered Gaussian, salt-and-pepper, and compound-disturbance results. Under Gaussian noise, C-KPFCM reaches average SA values of 0.6939, 0.7351, and 0.6444 at noise levels 0.01, 0.03, and 0.05, respectively. Under salt-and-pepper noise, it maintains SA values of 0.7637, 0.7515, 0.7410, and 0.7279 at densities 0.01, 0.03, 0.05, and 0.07, respectively. These values show that the method is especially effective when the corruption is sparse and local.

For a representative compound setting with simultaneous Gaussian and salt-and-pepper disturbance at (σ, density) = (0.03, 0.05), C-KPFCM obtains SA 0.7138, compared with 0.6984 for FCM and 0.5510 for PFCM. This result is consistent with the objective design: the cutset rule reduces the effect of corrupted pixels, while the kernel distance helps when the remaining class structure is not well represented by Euclidean prototypes.

The legacy materials also report broader stress-test behavior under illumination drift, block occlusion, texture clutter, and severe compound degradation. Those results narrow the claim. C-KPFCM remains strongest under clean and mixed-noise settings, but FCM can become slightly better under strong illumination drift and the most destructive compound scenario. Thus, the proposed method enlarges the useful robustness region for ambiguity and sparse contamination, but it does not eliminate all breakdown modes.

### 4.5 Parameter sensitivity and repeated-run stability

The recovered sensitivity analysis indicates that C-KPFCM is reasonably stable over a moderate parameter range, but the two parameters have different practical roles. Table 8 reports the parameter-sensitivity sweep for beta, sigma, and m. The cutset threshold is the more important robustness control: performance is highest at β = 0.5 and decreases when β is raised, because fewer non-winning typicalities are suppressed. The kernel width should be selected after feature normalization. In the reconstructed benchmark, σ = 0.18 gives the highest average SA, while σ = 0.22–0.30 remains close. We therefore use σ = 0.26 as a conservative default and recommend a small validation grid over {0.18, 0.22, 0.26, 0.30} when validation labels or trusted masks are available. The fuzzifier is less sensitive over *m*∈[1.5, 3.0], with the default *m* = 2 remaining competitive.

**Table 8:** Parameter-sensitivity summary for C-KPFCM on the reconstructed synthetic benchmark.

Parameter sweep	Average SA				
β: 0.50/0.60/0.70/0.80/0.90	0.7699	0.7697	0.7640	0.7064	0.6814
σ: 0.14/0.18/0.22/0.26/0.30	0.7439	0.7766	0.7715	0.7699	0.7693
*m*: 1.50/2.00/2.50/3.00	0.7656	0.7699	0.7664	0.7653	–

**Figure 8:**
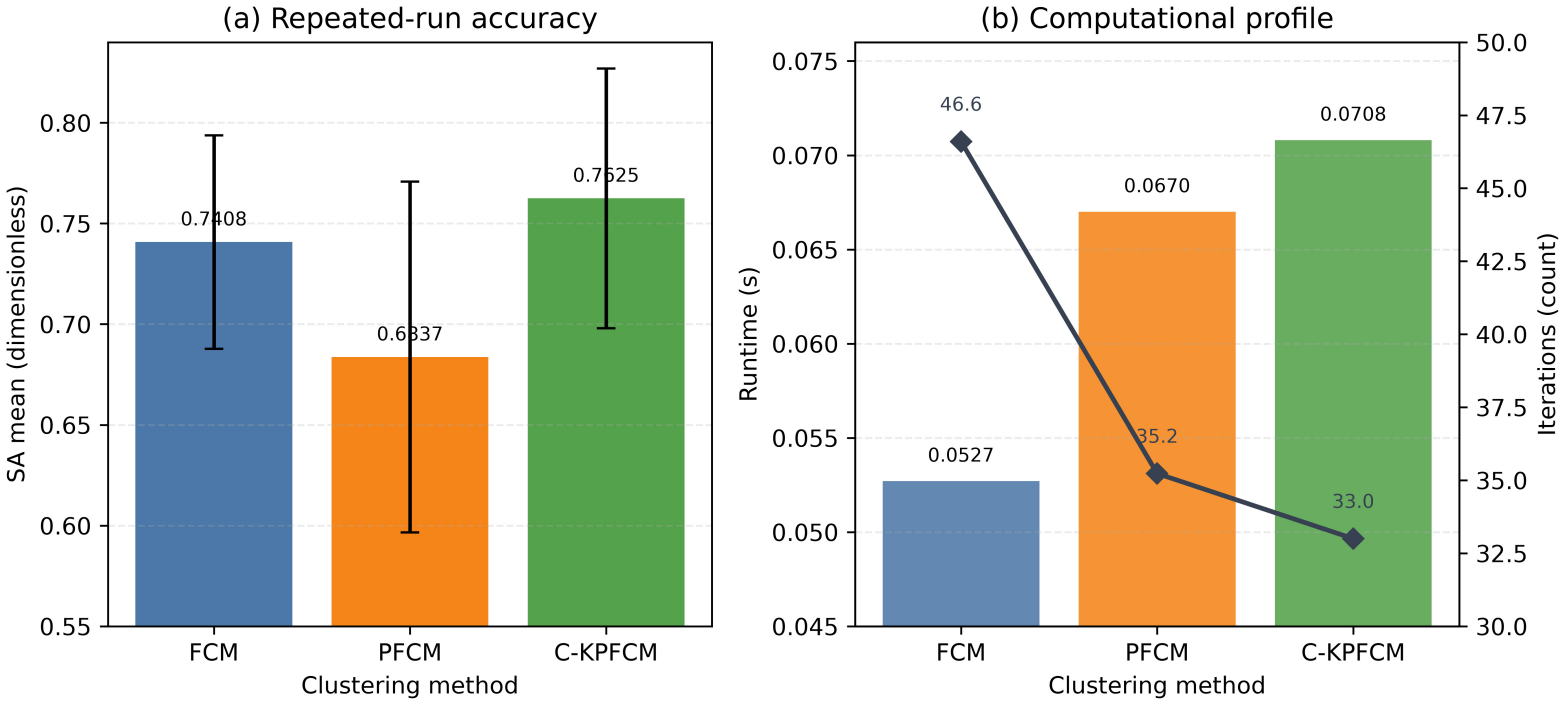
Repeated-run stability and computational profile over 10 random seeds. **(a)** C-KPFCM has the highest mean SA, with error bars indicating one standard deviation. **(b)** Runtime bars show that C-KPFCM is slower than FCM but close to PFCM, while the overlaid iteration curve shows that it uses the fewest average iterations in this recovered setting.

Table 9 and Figure 8 show that C-KPFCM improves repeated-run mean accuracy without a large runtime penalty relative to PFCM. Its average iteration count is lower than FCM in this experiment, while its runtime remains higher because of the kernel and typicality updates. The reported standard deviation of C-KPFCM, 0.0645 over 10 random seeds, is much larger than the 0.0016 mean difference between C-KPFCM and C-PFCM in Table 3. This again prevents a strong claim that kernelization is reliably better than cutset correction alone.

**Table 9:** Repeated-run stability and runtime comparison over 10 random seeds.

Method	SA mean	SA std	Iterations mean	Time mean (s)
FCM	0.7408	0.0530	46.60	0.0527
PFCM	0.6837	0.0871	35.23	0.0670
C-KPFCM	0.7625	0.0645	33.00	0.0708

### 4.6 Real-image validation

**Figure 9:**
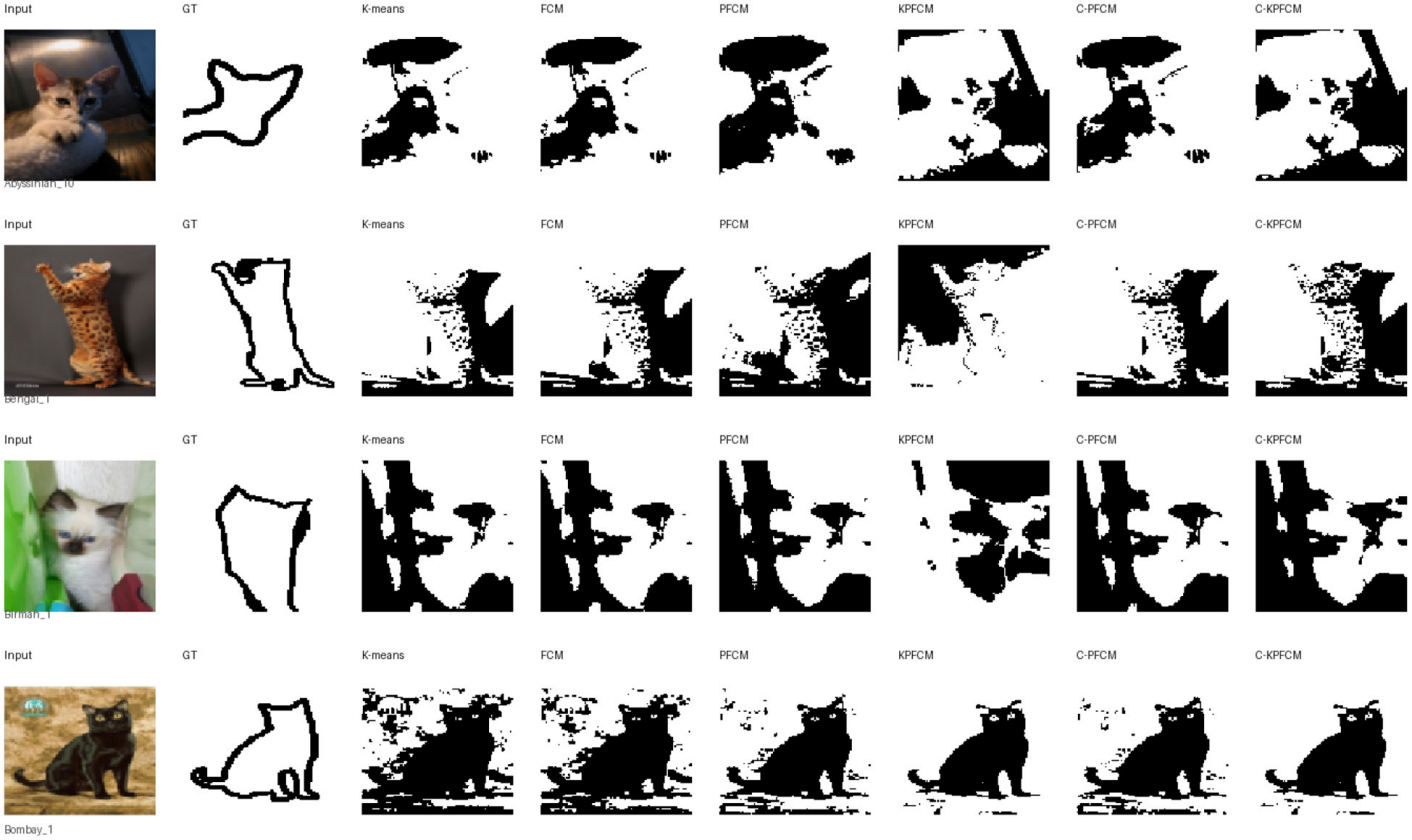
Representative qualitative outputs from the downloaded Oxford-IIIT Pet experiment (reproduced with permission from ‘The Oxford-IIT Pet Dataset', https://www.robots.ox.ac.uk/~vgg/data/pets/, created by Omkar M Parkhi, Andrea Vedaldi, Andrew Zisserman and C. V. Jawahar, licensed under CC BY-SA). The larger real-image test highlights the transfer boundary of the method family: textured natural backgrounds and binary foreground structure reduce the relative advantage of C-KPFCM, and simple color-spatial clustering baselines can be stronger.

To strengthen the real-data evidence during revision, the Oxford-IIIT Pet data were downloaded from the official release site and evaluated on 120 images with official trimaps converted to binary pet-vs-background masks. Table 10 reports the aggregate Oxford-IIIT Pet results, and Figure 9 shows representative Oxford-IIIT Pet outputs. The evaluated subset was sampled in a breed-balanced order from the successfully extracted image files. All images were resized to 96 × 96 and represented by RGB values plus normalized spatial coordinates. This larger real-image test remains a foreground-background clustering experiment rather than a full semantic-segmentation benchmark, but it is a substantially stronger check than the previous 12-image pilot.

**Table 10:** Downloaded Oxford-IIIT Pet real-image results on 120 images.

Method	SA	Dice	IoU	Time (s)
K-means	0.6151	0.7367	0.5884	0.0039
FCM	0.6122	0.7344	0.5852	0.0142
PFCM	0.5863	0.7088	0.5532	0.0253
KPFCM	0.5812	0.7062	0.5497	0.0533
C-PFCM	0.6022	0.7240	0.5724	0.0212
C-KPFCM	0.5829	0.7066	0.5506	0.0539

**Figure 10:**
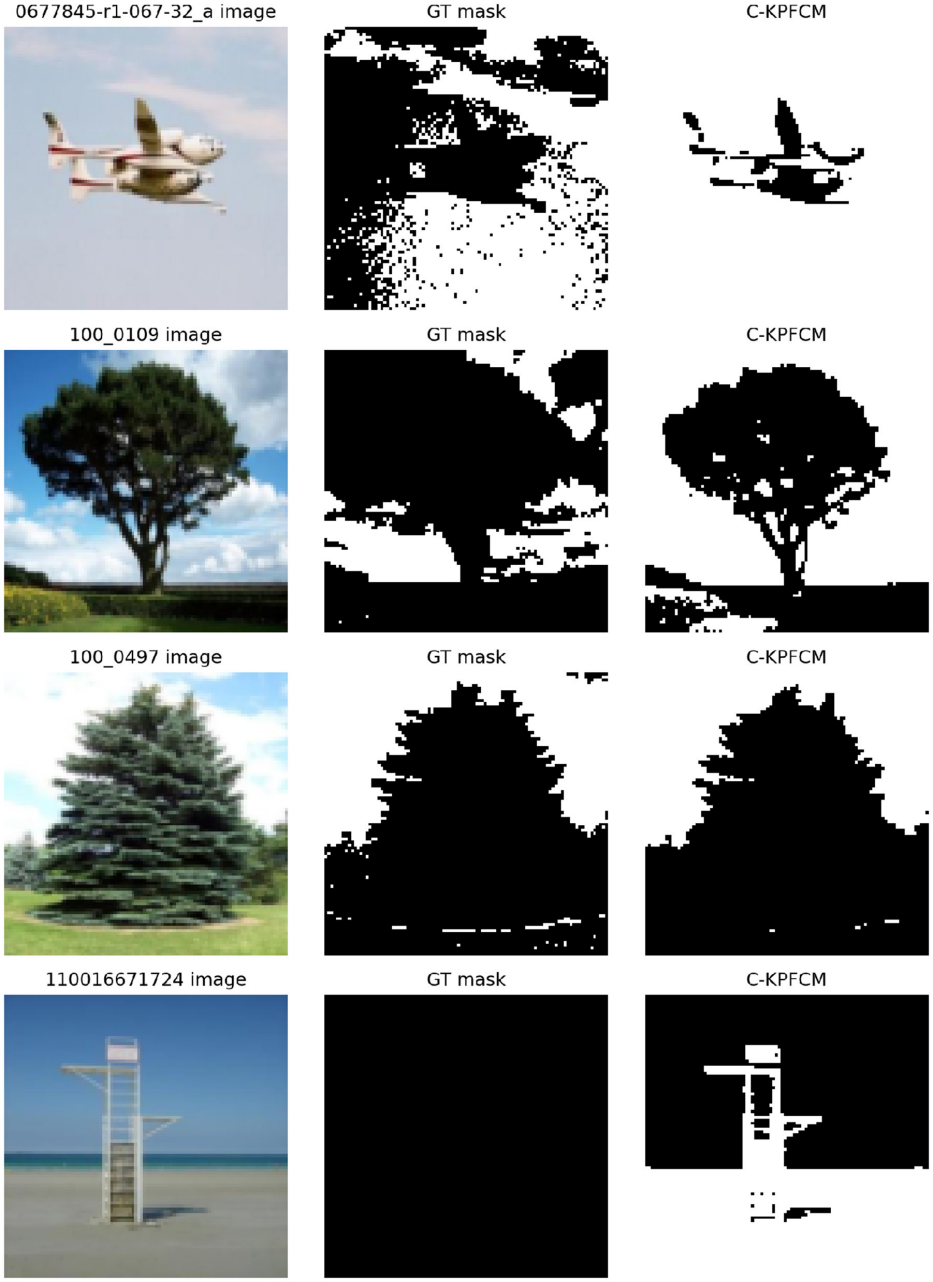
Representative Weizmann pilot outputs (images reproduced from the ‘Segmentation evaluation database', Weizmann Institute of Science, https://www.wisdom.weizmann.ac.il/vision/Seg_Evaluation_DB/dl.html). This exploratory pilot does not show a consistent advantage for C-KPFCM and is interpreted as boundary evidence rather than a main superiority result.

A second Weizmann single-object pilot is included as exploratory supplementary evidence because the source archive recovery is partial. Table 11 reports the exploratory Weizmann pilot results, and Figure 10 shows representative Weizmann outputs. The first 12 recoverable examples with color images and human segmentation masks were used in sorted order, each with three human masks. Original image sizes range from 300 × 170 to 300 × 407, and the foreground-pixel ratio ranges from 0.000 to 0.498, with mean 0.157. Several recovered masks are nearly empty, which lowers confidence in this pilot and is why it is not treated as a main benchmark.

**Table 11:** Exploratory Weizmann single-object pilot results.

Method	SA	Dice	IoU	Time (s)
FCM	0.6981	0.3285	0.2545	0.0150
PFCM	0.6631	0.3622	0.2871	0.0299
KPFCM	0.6784	0.3604	0.2925	0.0666
C-PFCM	0.6909	0.3674	0.2831	0.0264
C-KPFCM	0.6694	0.3498	0.2787	0.0781

The real-image experiments are scientifically useful because they are not uniformly favorable. On the downloaded Oxford-IIIT Pet subset, the synthetic ranking does not transfer: K-means and FCM are strongest on average, and C-PFCM is the strongest possibilistic variant. C-KPFCM remains close to KPFCM but is weaker than K-means, FCM, and C-PFCM in both mean Dice and the paired checks. The Weizmann pilot shows the same broad pattern, with low absolute Dice and IoU for all methods and no consistent C-KPFCM advantage. Table 12 confirms that the real-image checks do not support a superiority claim for C-KPFCM. These results clarify the appropriate scope of the method. C-KPFCM is most defensible for controlled settings with nonlinear overlap and sparse contamination, not as a general natural-image foreground-background solution.

**Table 12:** Paired real-image checks for C-KPFCM.

Dataset and metric	Reference	Mean difference	W/T/L	Sign-test *p*
Oxford SA	K-means	–0.0323	38/1/81	0.0001
Oxford SA	FCM	–0.0294	38/1/81	0.0001
Oxford SA	C-PFCM	–0.0193	42/2/76	0.0022
Oxford Dice	K-means	–0.0302	35/1/84	< 0.0001
Oxford Dice	FCM	–0.0278	34/2/84	< 0.0001
Oxford Dice	C-PFCM	–0.0174	40/2/78	0.0006
Weizmann SA	FCM	–0.0287	6/0/6	1.0000
Weizmann SA	C-PFCM	–0.0215	7/0/5	0.7744
Weizmann Dice	FCM	0.0212	4/5/3	1.0000
Weizmann Dice	C-PFCM	–0.0176	4/4/4	1.0000

Mean difference is C-KPFCM minus the reference method. W/T/L denotes image-level wins, ties, and losses for C-KPFCM; the sign test is exact and two-sided after removing ties.

## 5 Discussion

The experimental evidence supports three conclusions. First, cutset correction is the dominant contributor on the reconstructed synthetic benchmark and the expanded reviewer-requested stress benchmark. Both C-PFCM and C-KPFCM outperform KPFCM by a large margin, showing that suppressing non-winning typicalities is more important than kernelization alone for the tested contamination patterns.

Second, kernelization remains useful only as a conditional hypothesis supported by limited descriptive evidence. C-KPFCM achieves the best average SA on the three clean reconstructed synthetic cases and the best result on two of those cases, but the gain over C-PFCM is too small to survive the initial reliability check. In the expanded 90-instance stress test, C-KPFCM is slightly below C-PFCM on average and loses to C-PFCM in the paired sign test. The manuscript therefore treats kernelization as a conditional complement whose practical value depends on nonlinear geometry and must be verified before accepting the extra runtime cost.

Third, the method has clear boundary conditions. On WEI and on many noisy expanded instances, C-PFCM is better than C-KPFCM, suggesting that cutset correction alone can be sufficient when contamination dominates and nonlinear geometry is less important. Under real-image foreground-background pilots, the full synthetic ranking does not transfer. This does not invalidate the synthetic result; it specifies the use case more precisely. The method is a lightweight, reproducible option for disturbance-aware clustering segmentation, especially when the expected failure modes match its objective design, but it should not be presented as a broadly superior natural-image segmentation method.

## 6 Conclusion

This paper presents a revised and expanded study of a cutset-type kernel possibilistic fuzzy c-means method for robust image segmentation. By combining Gaussian-kernel distance with cutset-based typicality correction, C-KPFCM is designed for classical fuzzy clustering under nonlinear overlap, sparse local contamination, and complex background interference. On the three clean reconstructed synthetic cases, it achieves the highest average SA of 0.7699 and the best result on two of three cases. On the expanded 90-instance disturbance benchmark, however, C-KPFCM is clearly stronger than K-means, PFCM, and KPFCM but slightly weaker than C-PFCM on average.

The main scientific conclusion is mechanism-level rather than promotional. Cutset correction provides most of the observable robustness gain, while the incremental effect of kernelization over C-PFCM is small, disturbance-dependent, and not reliably positive in the current evidence. Retained real-image pilots on Oxford-IIIT Pet and Weizmann show that the synthetic advantage does not automatically transfer to generic natural-image foreground-background segmentation. Future work should therefore focus on larger annotated real-image validation, spatially regularized fuzzy baselines, recent lightweight unsupervised segmentation methods, and adaptive kernel selection for broader degradation regimes.

## Data Availability

The original contributions presented in the study are included in the article/supplementary material, further inquiries can be directed to the corresponding author.
